# Lansoprazole-Based Colorimetric Chemosensor for Efficient Binding and Sensing of Carbonate Ion: Spectroscopy and DFT Studies

**DOI:** 10.3389/fchem.2020.626472

**Published:** 2021-02-02

**Authors:** Mahdieh Darroudi, Ghodsi Mohammadi Ziarani, Shahriyar Bahar, Jahan B. Ghasemi, Alireza Badiei

**Affiliations:** ^1^Department of Chemistry, Faculty of Physics and Chemistry, Alzahra University, Tehran, Iran; ^2^School of Chemistry, College of Science, University of Tehran, Tehran, Iran

**Keywords:** lansoprazole, benzimidazole derivatives, chemosensor, carbonate ion, UV-Vis studies, density functional theory

## Abstract

The new benzimidazole based receptor **Lansoprazole** has been used to detect carbonate anion by naked-eye and Uv-Vis spectroscopy. This receptor revealed visual changes with ${\text{CO}}_{3}^{2-}$ anion in ethanol. No detectable color changes were observed upon the addition of any other tested anions. The lansoprazole chemosensor selectively recognizes ${\text{CO}}_{3}^{2-}$ ion over the other interference anions in the ethanol, followed by deprotonation and reflected 1:1 complex formation between the receptor and the carbonate ion. Lansoprazole exhibits splendid selectivity toward carbonate ion via a visible color change from colorless to yellow with a detection limit of 57 μM. The binding mode of ${\text{CO}}_{3}^{2-}$ to receptor **L** is supported by Density Functional Theory calculation. Moreover, this receptor shows a practical visible colorimetric test strip for the detection of carbonate ions. The transition states calculation demonstrates the occurrence of reaction from **L** to **L**-${\text{CO}}_{3}^{2-}$ after overcoming an energy barrier of 10.1 kcal/mol, and there is considerable interaction energy between **L** and ${\text{CO}}_{3}^{2-}$ (94.9 kJ/mol), both of which confirmed that receptor **L** has high sensitivity and selectivity to the carbonate ion. The theoretical studies were performed to acquire an electronic description of the complexation mechanism by ${\text{CO}}_{3}^{2-}$ as well as to study bonding and structure in the complex. The optimized structures and binding mechanisms were supported with a high correlation and agreement by spectroscopy and DFT calculations.

## Introduction

Developing anionic chemosensors is one of the most important topics nowadays. The optical sensor systems based on light usage as a most valuable and versatile output signal and several chromatographic and fluorogenic anion sensors have been recently established (Fabbrizzi et al., 2001, 2003; Ghorai et al., 2016a; Iqbal et al., 2018; Isaac et al., 2018; Wasim et al., 2018; Mahmudi et al., 2019; Mobed et al., 2019). Carbonate is one of these anions which is extensively used in the production of cosmetics, rubber, glass, printing ink, rayon, food, toothpaste, and rechargeable Li-ion batteries (Tas, 2009; Zhao et al., 2014; Ghorai et al., 2016a; Kordi et al., 2019; Darroudi et al., 2020). Moreover, carbonate has a pivotal role in agricultural planning, hydrology, soil, and geology science (Choi et al., 2002; Zougagh et al., 2005; Morris et al., 2010). Regardless of these broad applications in different industries, ${\text{CO}}_{3}^{2-}$ ion is a toxic chemical in high dosages as abdominal pain, collapse, and even death, thus developing a cost-effective and straightforward chemosensor for ${\text{CO}}_{3}^{2-}$ ion is vital (Jain et al., 2006; Abramova et al., 2010). Some analytical procedures have been established for ${\text{CO}}_{3}^{2-}$ ion detection, including continuous-flow, pH-ion sensitive field-effect transistor (Morf et al., 1989; Tsukada et al., 1990; Meruva and Meyerhoff, 1998), electrochemical (Lee et al., 2005), FT-IR spectroscopy (Burt and Rau, 1994), and chromo ionophore based optodes, etc. (Morf et al., 1989; Burt and Rau, 1994; Zougagh et al., 2005). However, these procedures are naked-eye-invisible and also time-consuming in most cases (García-Acosta et al., 2007; Movassagh et al., 2013; Ghorai et al., 2016a; Rouh et al., 2018; Smyth et al., 2020). While many probes have been described for the detection of various anions incorporate to acetate, sulfide, fluoride, and cyanide (Kondo and Takai, 2013; Reena et al., 2013; Tang et al., 2013, 2015; Zhang et al., 2013; Zheng et al., 2013; Kaur et al., 2015), limited chemosensors have been reported for ${\text{CO}}_{3}^{2-}$ ion (Hennrich et al., 2001; Rice, 2008; Han et al., 2010; Vaněk et al., 2013).

**Figure 1:**
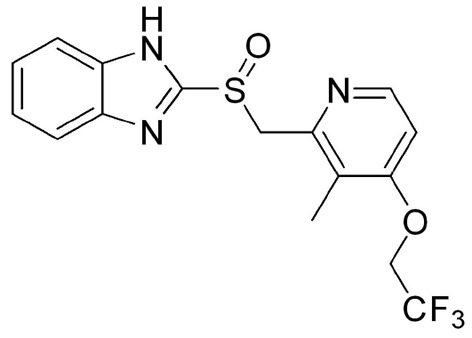
2-[[[3-methyl-4-(2,2,2,-trifluoroethoxy)-2-pyridil]sulfinyl-1H-benzimidazole(Lansoprazole).

On the other hand, one of the known aza heterocyclic structures in medicinal chemistry is benzimidazole scaffold, which has recently taken a great amount of attention in the scientific fields (Zhukova and Mamedov, 2017; Mostarda et al., 2019) because of their biological activities such as antibacterial (Xu et al., 2013), antifungal (Patel et al., 2014), antiviral (Monforte et al., 2008), antidiabetic (Liu et al., 2011), analgesic (Smith et al., 2008), and anticancer (Smith et al., 2008). Furthermore, benzimidazole-containing scaffolds, such as Lansoprazole currently is in use for the treatment of gastro-esophageal reflux and ulcer disease (Gremse, 2001; Miyashita et al., 2013; Shin and Kim, 2013; Yu et al., 2015; Estrada-Ortiz et al., 2019). The generic name of Lansoprazole is 2-[[[3-methyl-4-(2,2,2,- trifluoroethoxy)-2-pyridil] sulfinyl-1H-benzimidazole (Figure 1). To date, several fluorescents and UV-Vis sensors have been designed and reported, which some of them are benzimidazole-based, for various cations (Patel et al., 2013; Zhong et al., 2014; He et al., 2015; Maji et al., 2017; Liu C. et al., 2020) and anions (Yu et al., 2007; Zhang and Yu, 2017; Karuk Elmas et al., 2018; Ko et al., 2019; Liu F. et al., 2020; Tian and Li, 2020). Some carbonate chemosensors have been developed in recent years due to their rapidity, high sensitivity, and selectivity. However, some of the prepared sensors have much more detection limit to ${\text{CO}}_{3}^{2-}$ ion detection in high sensitivity, most of these chemosensors are difficult to prepare, or the reactions are not cost-effective (Ghorai et al., 2016b; Tavallali et al., 2016, 2019; Karuk Elmas et al., 2018; Kahriman et al., 2019; Naderi et al., 2019; Singh et al., 2019; Morikawa et al., 2020; Velmurugan et al., 2020). Herein, we proposed a naked-eye chemosensor, which has some superior as a simple process, biocompatibility, efficiency, side effects on the body, and rapid response time. As a part of our ongoing research on the design of chemosensors (Karimi et al., 2017; Shiravand et al., 2018, 2020; Ahmadi et al., 2019; Arab et al., 2019; Kordi et al., 2019), we report the study of a chemosensor (L) for efficient sensing of carbonate ion over other ions by UV-Visible spectroscopy and remarkable colorimetric responses in the solution. The chemosensor **L** detected ${\text{CO}}_{3}^{2-}$ ion by an alteration in absorbance accompanied by an instantaneous color change from colorless to yellow.

## Materials and Methods

### Materials and Instruments

All the commercial-grade chemicals and reagents and all organic solvents were purchased from Sigma-Aldrich company and were used without further purifications. Stock solutions of all metal ions were prepared using their nitrate salts purchasing from Merck company. Also, Stock solutions of all anions were prepared using their sodium salts purchasing from Merck company. All the UV-Vis absorption spectra were recorded on Analytik Jena Specord S600 Spectrophotometer using a 10 mm path length quartz cuvette.

### UV-Vis Studies

**Figure 2:**
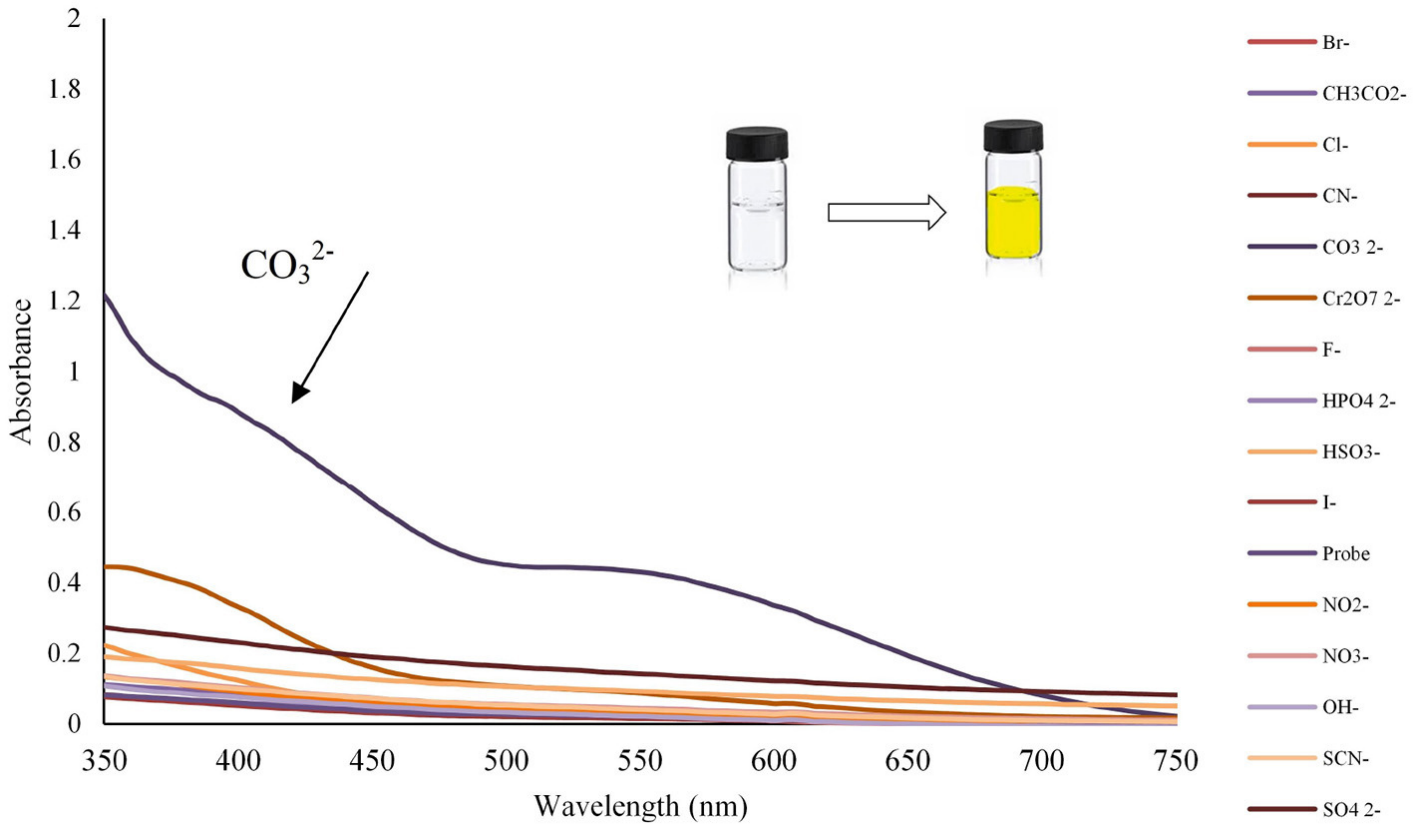
Change in absorption spectra and color changes of benzimidazole **L** (1 mL, 0.0001 M) after addition of different anions (100 μL, 0.01 M) in ethanol (pH = 10.2).

The anion recognition studies were performed at 25 ± 1 C, and before recording any spectrum, sufficient time was given to ensure the solution uniformity. The UV-Vis spectra of chemosensor **L** (1 × 10^−4^ mol L^−1^) probe was evaluated with the addition of different anions such as Br^−^, CH_3_COO^−^, Cl^−^, CN^−^, ${\text{CO}}_{3}^{2-}$, Cr_2_${\text{O}}_{7}^{2-}$, F^−^, ${\text{HPO}}_{4}^{2-}$, ${\text{HSO}}_{3}^{-}$, I^−^, ${\text{NO}}_{2}^{-}$, ${\text{NO}}_{3}^{-}$, OH^−^, SCN^−^, and SO4^2−^ with the concentration of 1 × 10^−2^ mol L^−1^. Among all studied anions, only carbonate ion could impose UV-Vis absorption of chemosensor **L**, which led to color change as a colorimetric sensor for ${\text{CO}}_{3}^{2-}$ ion, as shown in Figure 2. However, upon the ${\text{CO}}_{3}^{2}$, a strong red-shift on the absorption is observed; while, the other anions affect slightly.

### Interfering Anions

The sensor competition test of chemosensor **L** was investigated at 350 nm via the addition of ${\text{CO}}_{3}^{2-}$ (1 × 10^−2^ mol L^−1^, 100 μl) and other anions (1 × 10^−2^ mol L^−1^, 100 μl) to chemosensor **L** (1 mL, 1 × 10^−4^ mol L^−1^) transferring into a cell. After mixing them for a few seconds, the UV-Vis spectra were recorded at room temperature.

### Titration Experiments

The titration test was performed by adding the different concentration of carbonate ion (1 × 10^−2^ mol L^−1^) to chemosensor **L** (1 × 10^−4^ mol L^−1^). The mixture was transferred into a quartz cell. Then a certain amount of ${\text{CO}}_{3}^{2-}$ ion from 0 to 1,000 μl (1 × 10^−2^ mol L^−1^) was added to the chemosensor **L** probe solution. The UV-Vis absorption spectra were recorded after 1 min at room temperature.

### Computational

All calculations were carried out by DFT theory with B3LYP (Hay and Wadt, 1985) functionals as implemented in the Gaussian 09 program package (Robert, 1990). The geometries of all transition states (TSs) and ground states (GSs) were optimized utilizing 6-311+G(2d,2p) basis set (Hay and Wadt, 1985). Solvent effects were considered using CPCM calculations in the solvents (MacGregor, 1967; Luzar and Stefan, 1990; Klamt and Schüürmann, 1993; Andzelm et al., 1995; Barone and Cossi, 1998; Cossi et al., 2003; Jie and Guo-Zhu, 2013). On the basis of the optimized geometries, all energies were corrected with single point dispersion effect using the DFT-D2 method of Grimme (Grimme, 2006), as recent reports have shown that inclusion of these effects can significantly improve the accuracy of the B3LYP method (Xu et al., 2012).

## Results and Discussion

### UV-Vis Analysis

The detecting anion ability of Lansoprazole could be evaluated in ethanol by UV-Vis absorption spectra toward a wide range of anions including Br^−^, CH_3_COO^−^, Cl^−^, CN^−^, ${\text{CO}}_{3}^{2-}$, Cr_2_${\text{O}}_{7}^{2-}$, F^−^, ${\text{HPO}}_{4}^{2-}$, ${\text{HSO}}_{3}^{-}$, I^−^, ${\text{NO}}_{2}^{-}$, ${\text{NO}}_{3}^{-}$, OH^−^, SCN^−^, and SO4^2−^at 350 nm (Figure 2). The spectra were instantly recorded after ion addition to Lansoprazole, upon absorbance between 200 and 750 nm, which strongly increased at 300–700 nm in the presence of carbonate ion. Upon adding carbonate ion to Lansoprazole, distinct spectral changes were induced, and a red-shift in the absorbance spectra was observed, while the other ions display no tangible changes along with the color change from colorless to yellow. These results show that Lansoprazole can be attended as a naked-eye ${\text{CO}}_{3}^{2-}$ detector.

### Interfering Anions

**Figure 3:**
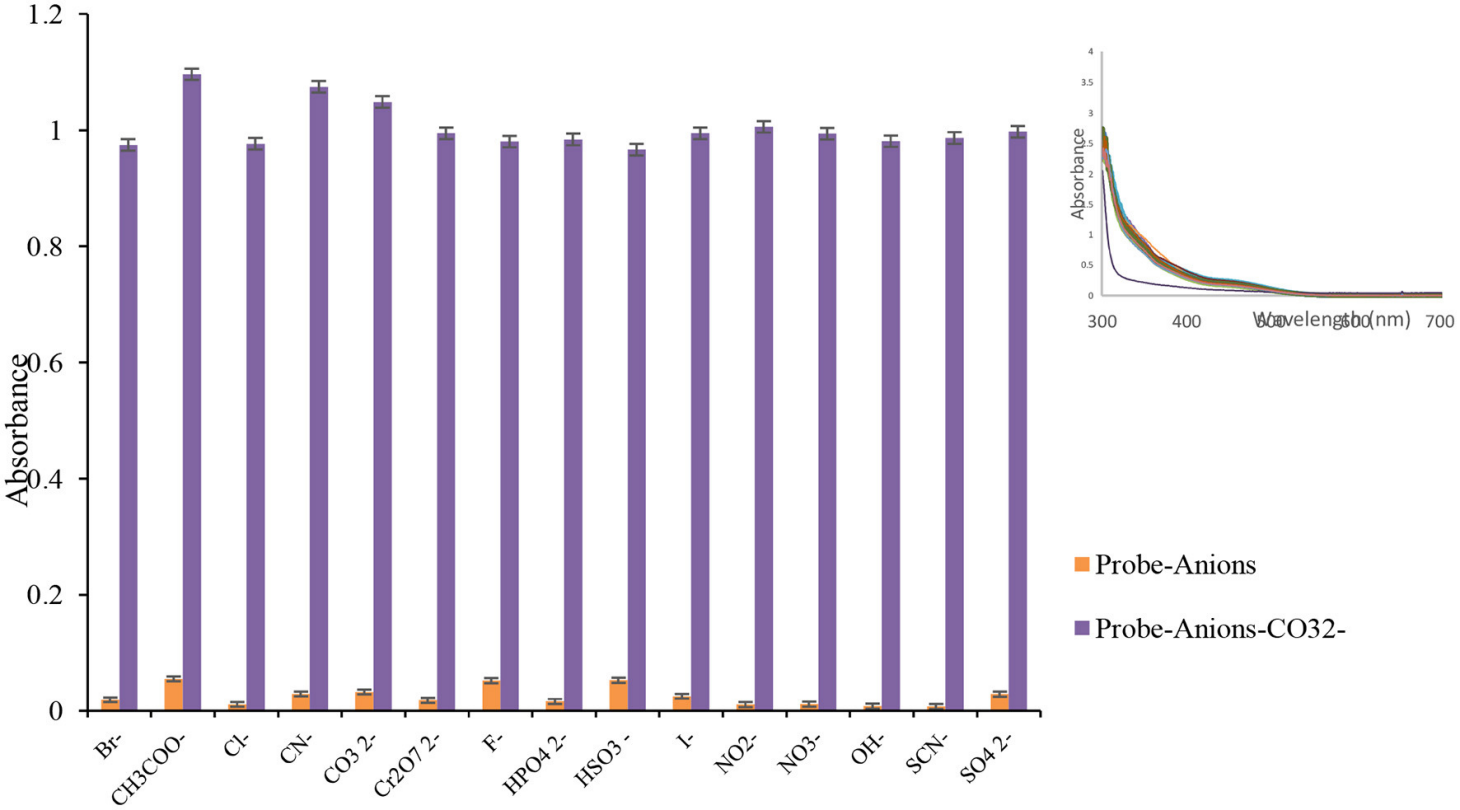
The competitive experiment of chemosensor L (100 μL, 0.01 M) ${\text{CO}}_{3}^{2-}$ ion at 480 nm in the presence of other anions (100 μL, 0.01 M), and inset: absorbance spectra of probe solution (1 mL, 0.001 M) after addition of various anions (100 μL, 0.01 M). *n* = 3, Sd _average_ = 1.86.

To investigate the selectivity of the prepared probe, the consistent mixtures of target ions and common interfering anions were correspondingly checked, and the results are represented in Figure 3. The absorbance spectra of chemosensor **L** were monitored in ${\text{CO}}_{3}^{2-}$ ion and equivalent amounts of competing anions. The ${\text{CO}}_{3}^{2-}$ ion created a significant red shift in Lansoprazole's UV-Vis spectra than other ions, as shown in Figure 3.

To determine the influence of other anions on the detection of carbonate anion in ethanol, different anions with a concentration of 0.01 M were added to the Lansoprazole (1 × 10^−4^ mol L^−1^) containing ${\text{CO}}_{3}^{2-}$ (1 × 10^−2^ mol L^−1^). It was exhibited that other anions under experiment did not interfere with the detection of carbonate ion by receptor **L**. The interfering ions induced small or no tangible changes in the absorbance system. As a result, this probe can be considered as a highly selective and reliable probe for ${\text{CO}}_{3}^{2-}$ ion detection.

### Titration Experiments

**Figure 4:**
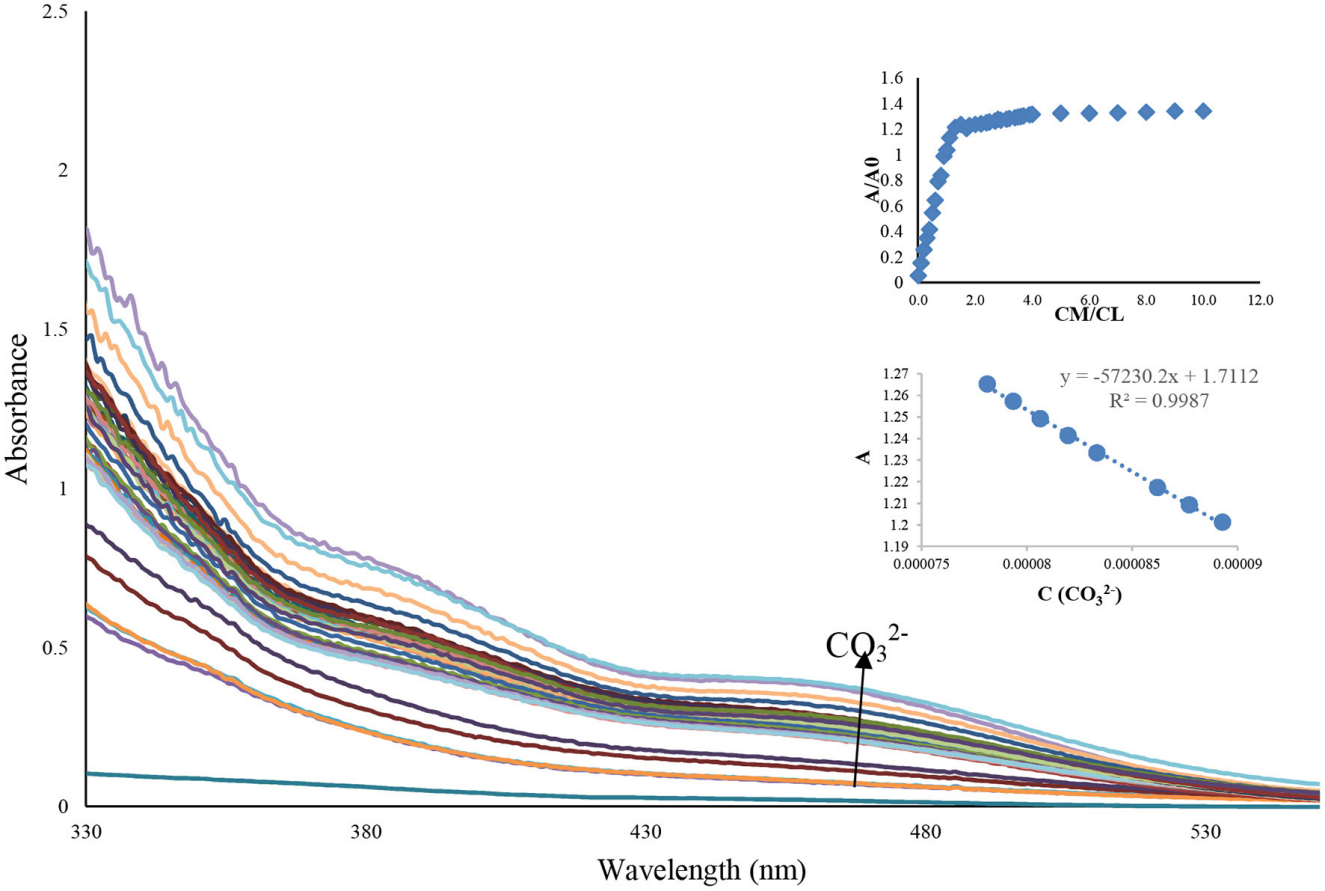
Absorbance spectra of Benzimidazole **L** (1 mL, 0.0001 M) after adding the different concentration of an aqueous solution of ${\text{CO}}_{3}^{2-}$ ion (10–1,000 μL, 0.01 M), inset: the relationship between the concentration of ${\text{CO}}_{3}^{2-}$ and maximum absorbance at 480 nm.

To evaluate the interacting potential of probe and ${\text{CO}}_{3}^{2-}$, the titration experiments were performed (Figure 4).

Chemosensor **L** showed by the increase of carbonate ion concentration from 0 to 20 eq, all the absorption peaks at 480 nm were enhanced significantly. In the UV-Vis titration experiment, the red-shifted band triggered by carbonate ion continuously increases with the successive increment of carbonate ion (0–10 eq) into the chemosensor **L**, as shown in Figure 4. The inset in Figure 4 is a plot of the UV-Vis absorption of chemosensor **L**. It can be seen that absorption increases through the increase of ${\text{CO}}_{3}^{2-}$ from 0 to 10 μM. When the concentration of ${\text{CO}}_{3}^{2-}$ is larger than 100 μM, the absorption spectra almost remains unchanged. The detection limit of Lansoprazole for ${\text{CO}}_{3}^{2-}$ was determined to be 0.57 × 10^−6^ M with a linear range of concentration to be 0–10 μM. The apparent bathochromic shift that occurred by carbonate ion led us to propose that the π conjugate system of chemosensor **L** underwent intramolecular charge transfer from donor to acceptor by excitation through the deprotonation of sensor **L** by ${\text{CO}}_{3}^{2-}$ ion. Lansoprazole's ratiometric response on carbonate addition indicated a 1:1 stoichiometric adduct of chemosensor **L** and carbonate ion (Figure 4). After concentration enhancement of ${\text{CO}}_{3}^{2-}$ ion from 100 to 1,000 μM, the absorbance reaches a plateau at 390 nm, while the absorbance is steadily (insets in Figure 4). To identify the charge transfer of chemosensor **L**, we have checked the change of its absorption spectra in different solvents such as DMSO, MeOH, H_2_O: EtOH, and Acetonitrile since it has been reported that the dipole of solvents can relax the charge transfer excited by polar solvents. As summarized in Table 1, the absorption spectra of chemosensor L featured a marginal absorption red-shift maximum at 11 nm, which indicate an apparent solvent dependence of the absorption band. To confirm whether the color change and absorbance change occur due to charge transfer due to the deprotonation mechanism. Therefore, the solvatochromic behavior demonstrated the occurrence of the charge transfer transition in chemosensor **L**. The fact that the sensing of carbonate ion by receptor **L** does not depend on counter metal ion, established by a similar type of absorbance spectra demonstrated by **L** with potassium carbonate.

**Table 1:** Absorption properties of chemosensor L in various solvents.

**Solvent**	**λ_abs_ (nm)**
MeOH	473
Acetonitrile	482
H_2_O: EtOH	476
DMSO	485

### Application of Probe L

**Figure 5:**
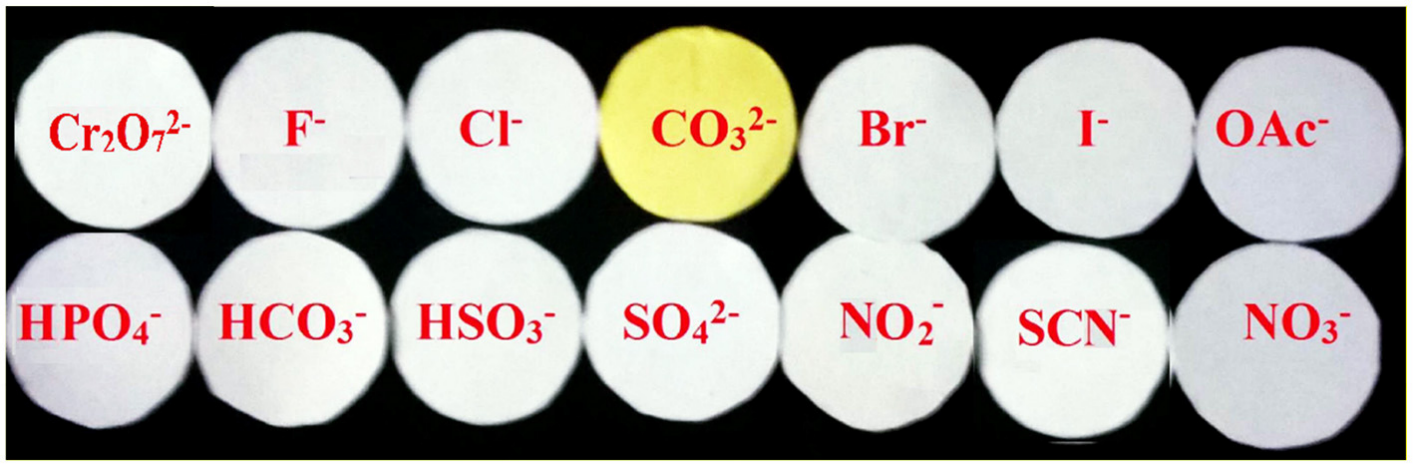
Photographs of the lansoprazole test kits (0.5 mM) in order to detect ${\text{CO}}_{3}^{2-}$ rather than other anions.

To check the practical applications, the test kits were utilized to sense ${\text{CO}}_{3}^{2-}$ among different competing anions. As shown in Figure 5, When the test kits coated with chemosensor **L** were added to different anion solutions, the distinctive color change was detected only with ${\text{CO}}_{3}^{2-}$ in ethanol solution. Therefore, the test kits coated with the probe **L** solution would be convenient for detecting carbonate. These results depicted that chemosensor **L** could be a valuable practical chemosensor for the analysis of carbonate ions.

### Reversibility

**Figure 6:**
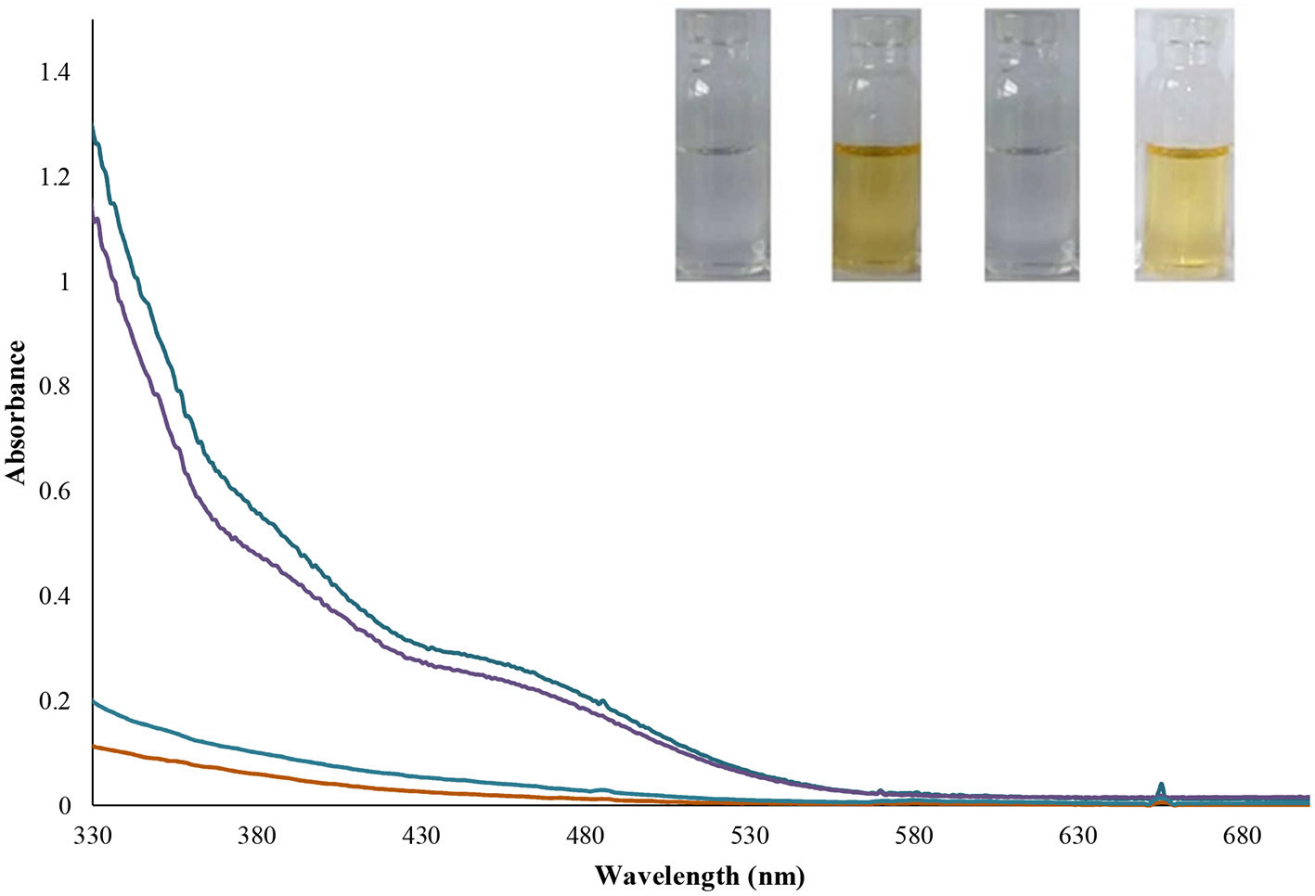
Reversibility test of chemosensor L with acetic acid and sodium carbonate addition.

The reversibility of the receptor lansoprazole was performed by adding acetic acid and Na^+^ binding agent. The addition of acetic acid to a mixture of chemosensor L and sodium carbonate resulted in the appearance of a peak at 480 nm, which indicates the regeneration of the receptor L. The absorption band at 480 nm was recovered by the addition of sodium carbonate, the same as the first. Such reversibility is vital for the fabrication of devices to sense the ${\text{CO}}_{3}^{2-}$ ion (Figure 6).

### Theoretical Study

**Figure 7:**
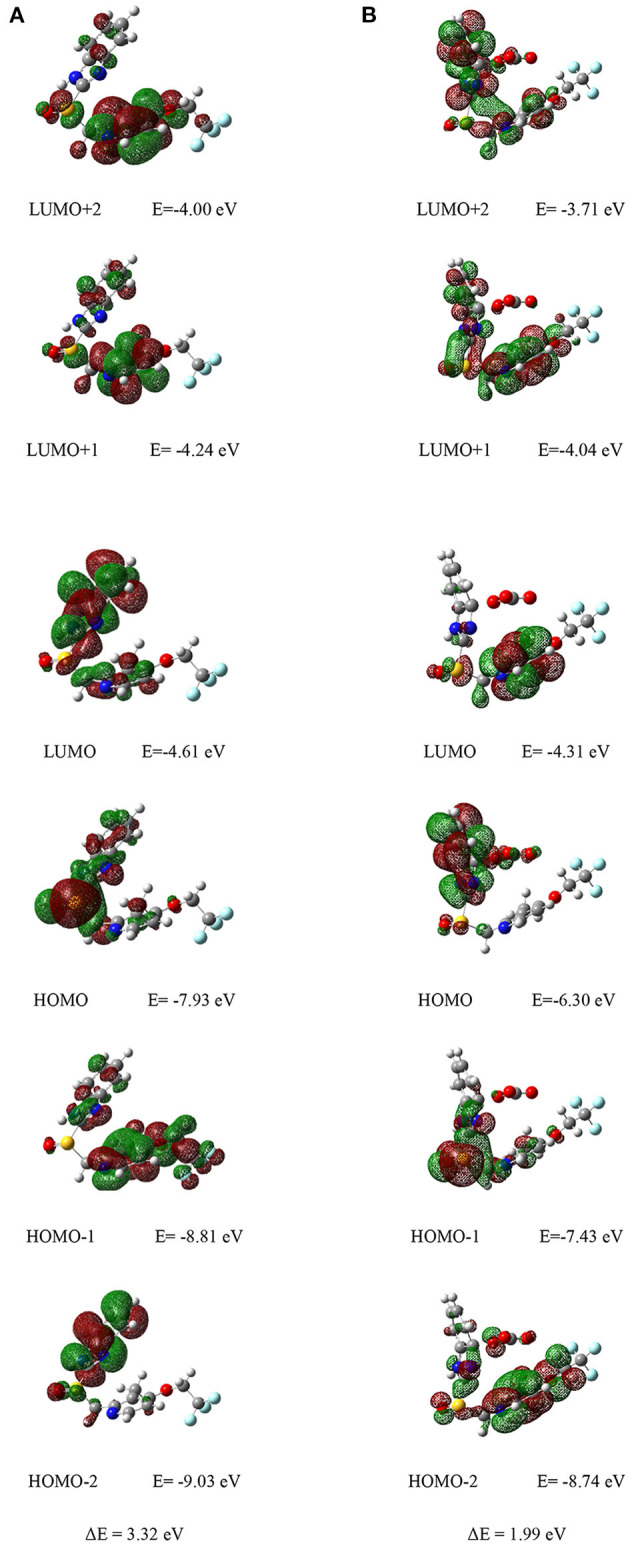
**(A)** Corresponding orbital electron distribution LUMO+2, LUMO+, LUMO, HOMO, HOMO-1, and HOMO-2 of **(A)** chemosensor **L** and **(B)** complex L-${\text{CO}}_{3}^{2-}$.

The molecular orbital (MO) energy level and distribution of the Lansoprazole was carried out by density functional theory (DFT) calculation at the B3LYP/6-311+G(2d,2p) level (Ishtiaq et al., 2016; Iqbal et al., 2018; Isaac et al., 2018; Islam et al., 2018). As we can see from Figure 7, the electron clouds of LUMO+1, LUMO+2 level of energy for chemosensor **L** were mainly distributed on the pyridine, and for LUMO level cloud was distributed on the C=N bond of benzimidazole group. While the electron density of HOMO was delocalized over the sulfoxide group, the electron density of HOMO-1 was almost populated on the C=N bond of the pyridine group, and the electron clouds of HOMO-2 were concentrated on the nitrogen in a five-membered ring. Therefore, transitions between all levels of energy had occurred for Lansoprazole. These levels of energies are much accessible for electron transfer.

Furthermore, the orbital energies of Lansoprazole were sequentially increased (Figure 7B), which indicates the easy coordination ability of chemosensor toward ion. Also, the electron density of LUMO+2 and HOMO of complex L-${\text{CO}}_{3}^{2-}$ were mainly populated on the benzimidazole group (Figure 7B). While the electron density of LUMO+1 and HOMO-2 were gathered together in the pyridine ring, the electron density of LUMO, HOMO-1 were mainly concentrated on the sulfoxide group. The distribution and MO level of energy indicated the complexation of Lansoprazole and ${\text{CO}}_{3}^{2-}$ ion through intermolecular charge transfer (CT). Notably, the calculated energy gaps between HOMO and LUMO decreased, respectively: 1.99 <3.32 for probe **L** and **L**-${\text{CO}}_{3}^{2-}$ complex. Such lowering HOMO-LUMO gaps of Lansoprazole upon ${\text{CO}}_{3}^{2-}$ complexation attributed to the electron distributions after bindings. The electron redistributions resulted in the absorbance change with subsequent shifts.

**Figure 8:**
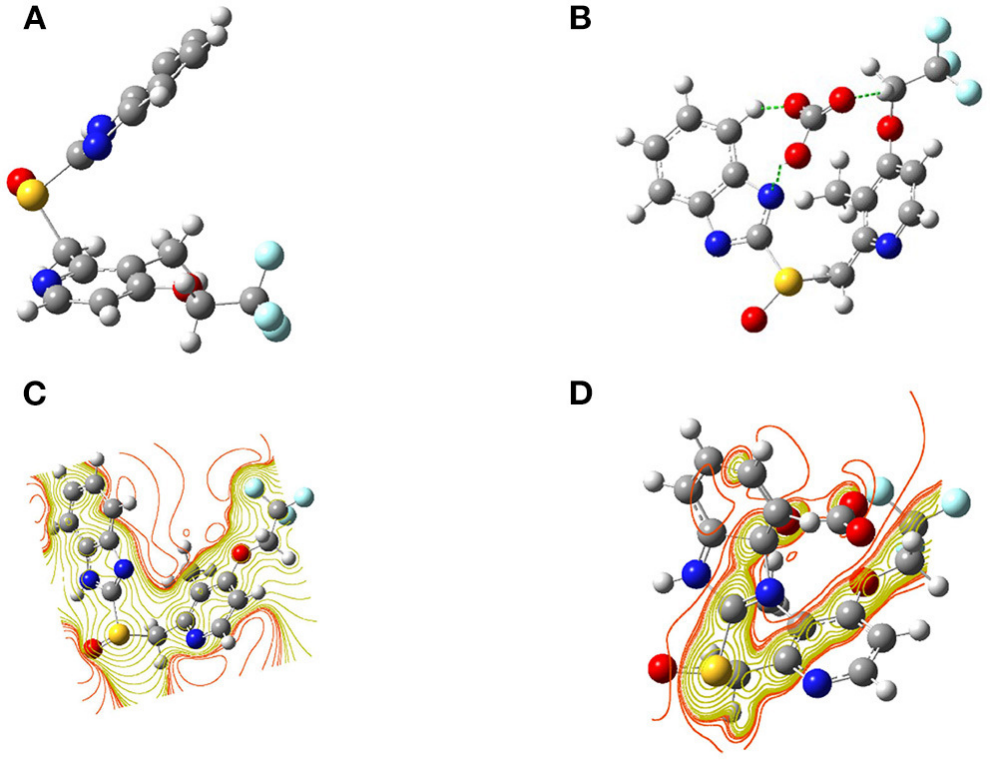
Optimized geometries of chemosensor **(A) L**, and **(B) L**-CO_3_^**2−**^ and also the SCF counter of **(C) L** and **(D) L**-${\text{CO}}_{3}^{2-}$.

Computations on the probe **L**, both after and before combination with ${\text{CO}}_{3}^{2-}$ were investigated in order to get the close approach toward the possible binding mechanism. The optimized structures and a schematic representation of SCF counters have been shown for probe **L** and **L**-${\text{CO}}_{3}^{2-}$ complex in Figure 8. The SCF counter showed that the chemosensor **L** had a uniform electron cloud scattering throughout the molecule and some active sites on sulfoxide, pyridine, and benzimidazole groups shown in Figure 6. Otherwise, in **L**-${\text{CO}}_{3}^{2-}$ complex, a strong electron distribution was changed entirely, and the electron density distribution is completely altering from chemosensor **L**. Moreover, a strong interaction was exhibited between the benzimidazole group, CH_2_ group linked to CF_3_, and ${\text{CO}}_{3}^{2-}$ anion.

### Mulliken Charge Analysis

Mulliken charge analysis was done through the calculation of the electron population for an individual atom. In chemosensor **L**, the positive charge strongly found at S10, and moderately on C4, C15, and C22. The positive charge for the sulfur atom is because of electronegative atom O and C atom because of conjugated structure in the closest sites. The O19 atom has negative charges; C9 atom attached to S atom possesses a negative charge. MEP is allied to electron density and is a very suitable descriptor in acceptor sites for the nucleophilic and electrophilic attack as hydrogen bonding interactions. The positive electron density localized on the S atom and C atoms of benzimidazole and pyridine structures.

### Mechanism of Sensing

**Figure 9:**
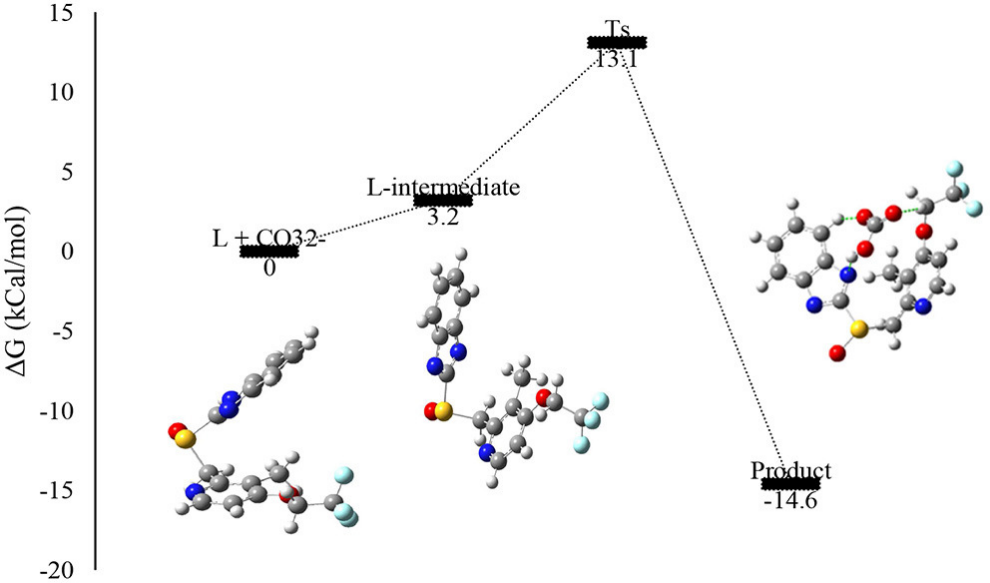
Free energy profile for the nucleophilic addition reaction of chemosensor **L** and ${\text{CO}}_{3}^{2-}$.

To elucidate the interaction process of the chemosensor **L** and ${\text{CO}}_{3}^{2-}$ ion, the Gibbs free energy profile of the reaction is calculated, displayed in Figure 9. We choose the reactant energies as the zero-point energy, and the data are listed in Figure 9. The formation of the intermediate complex is carried out by absorbing the energy of 3.2 kcal/mol and a transition states is found by TS theory, which is located at the cleavage of C6, C10, and S8 and the formation of ${\text{CO}}_{3}^{2-}$-S and ${\text{CO}}_{3}^{2-}$-C bonds. In addition, through the IRC calculation, we have confirmed that the transition state is indeed linked to the intermediate and product. The reaction barrier from the intermediate complex to the TS is 10.1 kcal/mol, which is relatively low. The low reaction barrier means that the chemosensor **L** has a favorable response speed to ${\text{CO}}_{3}^{2-}$ ion.

To explore the selectivity of the chemosensor L to carbonate, the interaction energies between chemosensor L and different anions have been studied, which are listed in Table 2. We calculated the interaction energies based on the E_int_ = E_L_ +E_anion_ – E_p_. As seen in Table 2, the interaction energy between **L** and ${\text{CO}}_{3}^{2-}$ is the largest one among these energies. The considerable interaction energy confirms the excellent selectivity of the chemosensor **L** for ${\text{CO}}_{3}^{2-}$.

**Table 2:** Calculated interaction energies for the chemosensor **L** with different anions.

**Anions**	**E_**int**_ (kJ/mol)**
${\text{CO}}_{3}^{2-}$	94.9
F^−^	72.6
Cl^−^	34.1
Br^−^	22.9
I-	36.7
SCN^−^	22.3
AcO^−^	29.5
NO_3_-	11.3
${\text{NO}}_{2}^{-}$	17.5
Cr_2_${\text{O}}_{7}^{2-}$	40.9
${\text{HPO}}_{4}^{-}$	14.2
${\text{SO}}_{4}^{2-}$	38.6
${\text{HSO}}_{3}^{-}$	30.7
OH^−^	69.1

## Conclusion

In summary, the receptor Lansoprazole has shown the colorimetric response in the presence of ${\text{CO}}_{3}^{2-}$ ion in ethanol solution with high degree of selectivity. This naked-eye colorimetric chemosensor for the detection of carbonate ion revealed a low detection limit 0.57 μM. It has excellent sensitivity and selectivity to sense ${\text{CO}}_{3}^{2-}$ by changes in absorption over a variety of anions. Furthermore, the binding mechanism of the Lansoprazole toward ${\text{CO}}_{3}^{2-}$ ions was investigated by DFT calculation. The structural parameter analysis indicates that the intramolecular bonding in chemosensor **L** and the ${\text{CO}}_{3}^{2-}$ ion, which induce the intramolecular proton transfer. The Ts calculation demonstrates that the reaction between L and ${\text{CO}}_{3}^{2-}$ need overcoming a small energy barrier (10.1 kcal/mol) and the interaction energy (94.9 kJ/mol), both of which confirmed that chemosensor L has high sensitivity and selectivity to ${\text{CO}}_{3}^{2-}$ ion. The experiments' results and theoretical works beside each other can be sued to the chemosensor's development and clarify the mechanism of sensing metal ions, anions, and drug molecules.

## Data Availability Statement

The original contributions presented in the study are included in the article/Supplementary Materials, further inquiries can be directed to the corresponding author/s.

## Author Contributions

MD: visualization, formal analysis, computational study, analysis data, and writing original draft. GM: funding acquisition, methodology, supervision, and writing—review and editing. SB: visualization, methodology, analysis data, and writing—review and editing. JG: methodology and writing—review and editing. AB: writing—review and editing. All authors contributed to the article and approved the submitted version.

## Conflict of Interest

The authors declare that the research was conducted in the absence of any commercial or financial relationships that could be construed as a potential conflict of interest.
