# Supplementary material for: Lansoprazole-Based Colorimetric Chemosensor for Efficient Binding and Sensing of Carbonate Ion: Spectroscopy and DFT Studies
Source: Front Chem. 2021 Feb 2;8:626472. doi: 10.3389/fchem.2020.626472 (PMC7884336; doi:10.3389/fchem.2020.626472)
Supplement: Supplementary file 1 [file Data_Sheet_1.docx]

Supplementary Material

Lansoprazole as a Selective Colorimetric Carbonate Chemosensor: Spectroscopy and DFT Studies

Mahdieh Darroudi, ^1,*^ Ghodsi Mohammadi Ziarani,^1,*^ Shahriyar Bahar,^1^ Jahan B. Ghasemi,^2^ Alireza Badiei^2^

^1^ Department of Chemistry, Faculty of Physic and Chemistry, Alzahra University, Tehran, Iran. P.O. Box 1993893973

^2^School of Chemistry, College of Science, University of Tehran, Tehran, Iran*****

**Correspondence:**Mahdieh Darroudi, ^1,*^ Ghodsi Mohammadi Ziarani,^1,*^
[M.darroudi@alzahra.ac.ir](mailto:M.darroudi@alzahra.ac.ir), [gmohammadi@alzahra.ac.ir](mailto:gmohammadi@alzahra.ac.ir)

**Materials**

All the commercial-grade chemicals and reagents and all organic solvents were purchased from Merck company.

- 1. **Materials and instruments**

All the commercial-grade chemicals and reagents were used without further purifications. Stock solutions of all metal ions were prepared using their nitrate salts purchasing from Merck company. Also, Stock solutions of all anions were prepared using their sodium salts purchasing from Merck company. All the UV-Vis absorption spectra were recorded on Analytik Jena Specord S600 Spectrophotometer using a 10 mm path length quartz cuvette.

- 1. **Probe preparation:**
- Lansoprazole **L** (369.3 mg, 1 mmol) was dissolved in ethanol (100 ml). The stock solution (1 × 10^3^ µl) was diluted to 100 ml by ethanol to prepare the final concentration of chemosensor **L** probe (1 × 10^-4^ mol L^-1^).
  1. **Anion preparation**

The stock solutions of various anions such as Br^-^, CH_3_COO^-^, Cl^-^, CN^-^, CO_3_^2-^, Cr_2_O_7_^2-^, F^-^, HPO_4_^2-^, HSO_3_^-^, I-, NO_2_^-^, NO_3_^-^, OH^-^, SCN^-^, and SO4^2-^ (1 × 10^-2^ mol L^-1^) were prepared by dissolving a suitable amount of sodium salts in double-distilled pure water. For anion sensing experiments, 100 μl of each ion (1 × 10^-2^ mol L^-1^) mixed with the chemosensor **L** solution (1 mL), which were used for UV-Vis absorption analysis.

**Fig. S1.** The detection limit of absorbance spectra for Benzimidazole L (1 ml, 0.0001 M) after addition of different concentration of an aqueous solution of CO_3_^2-^ ion at 350 nm


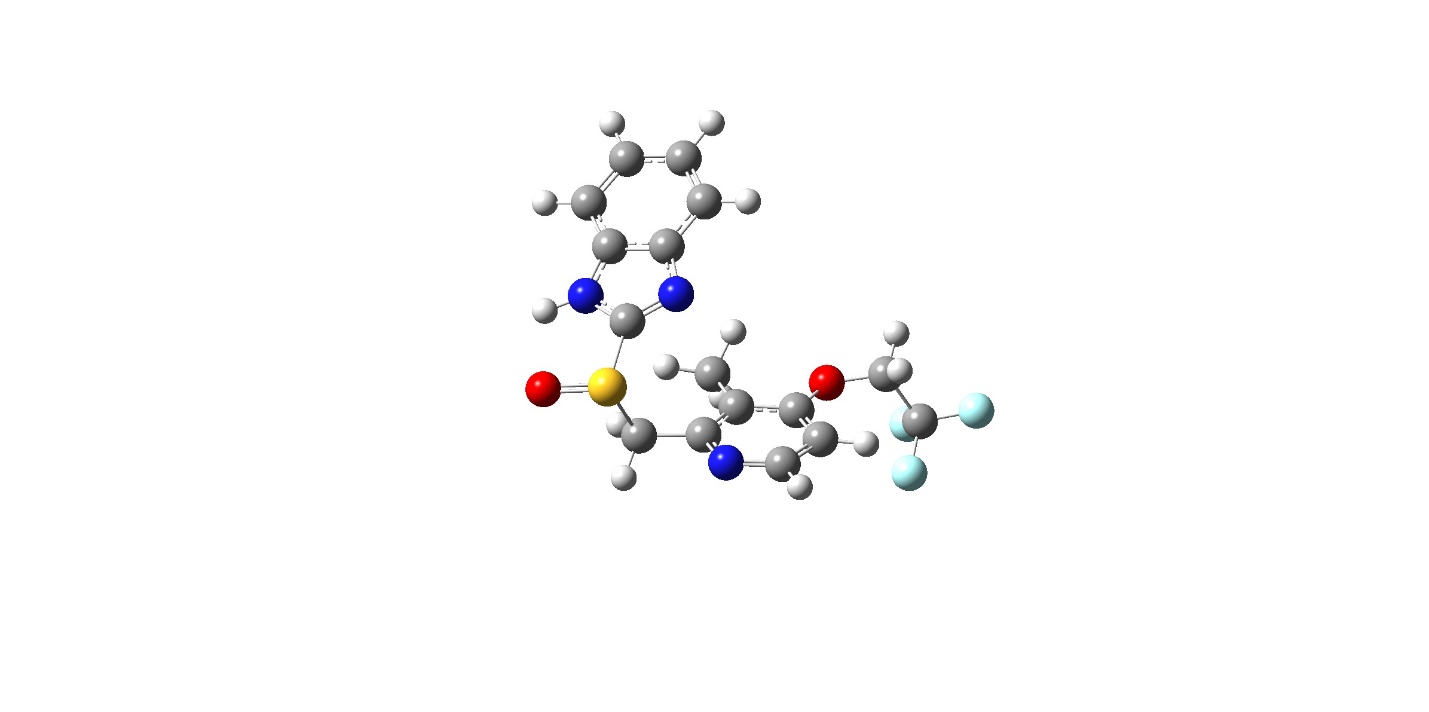


**Fig. S2.**

***** Axes restored to original set *****

orientation:

---------------------------------------------------------------------

Center Atomic Atomic Coordinates (Angstroms)

Number Number Type X Y Z

---------------------------------------------------------------------

1 6 0 -3.262592 4.068603 -0.139257

2 6 0 -2.284356 4.019237 0.875042

3 6 0 -1.795046 2.810988 1.351220

4 6 0 -2.304123 1.632833 0.791293

5 6 0 -3.285099 1.700706 -0.229036

6 6 0 -3.781431 2.913340 -0.710000

7 7 0 -2.008325 0.304796 1.083245

8 6 0 -2.764064 -0.374476 0.263166

9 7 0 -3.555311 0.384099 -0.547648

10 16 0 -2.919448 -2.174220 0.181267

11 8 0 -4.152466 -2.380354 -0.690738

12 6 0 -1.462218 -2.530106 -0.940572

13 6 0 -0.140653 -2.316408 -0.260421

14 6 0 0.735404 -1.281160 -0.602807

15 6 0 1.948674 -1.225112 0.115157

16 6 0 2.221186 -2.150179 1.116529

17 6 0 1.258883 -3.123800 1.372674

18 7 0 0.111697 -3.220434 0.709708

19 6 0 0.489341 -0.245111 -1.667672

20 8 0 2.788410 -0.219824 -0.248158

21 6 0 4.031164 -0.086303 0.420796

22 6 0 4.725664 1.110135 -0.197036

23 9 0 5.921241 1.296623 0.398226

24 9 0 4.012635 2.243316 -0.048594

25 9 0 4.945470 0.944694 -1.515677

26 1 0 -3.618054 5.033126 -0.482576

27 1 0 -1.909505 4.947783 1.289495

28 1 0 -1.044417 2.769369 2.131297

29 1 0 -4.532326 2.952400 -1.489302

30 1 0 -4.231549 0.006340 -1.197874

31 1 0 -1.604834 -3.591531 -1.147149

32 1 0 -1.632247 -1.956104 -1.847077

33 1 0 3.138110 -2.135433 1.688606

34 1 0 1.434948 -3.861474 2.149822

35 1 0 1.259599 -0.301162 -2.441950

36 1 0 -0.480553 -0.353647 -2.146078

37 1 0 0.545780 0.759092 -1.240070

38 1 0 3.898674 0.105309 1.488616

39 1 0 4.665067 -0.965190 0.277951

---------------------------------------------------------------------

Cartesian Forces: Max 0.000030451 RMS 0.000009755


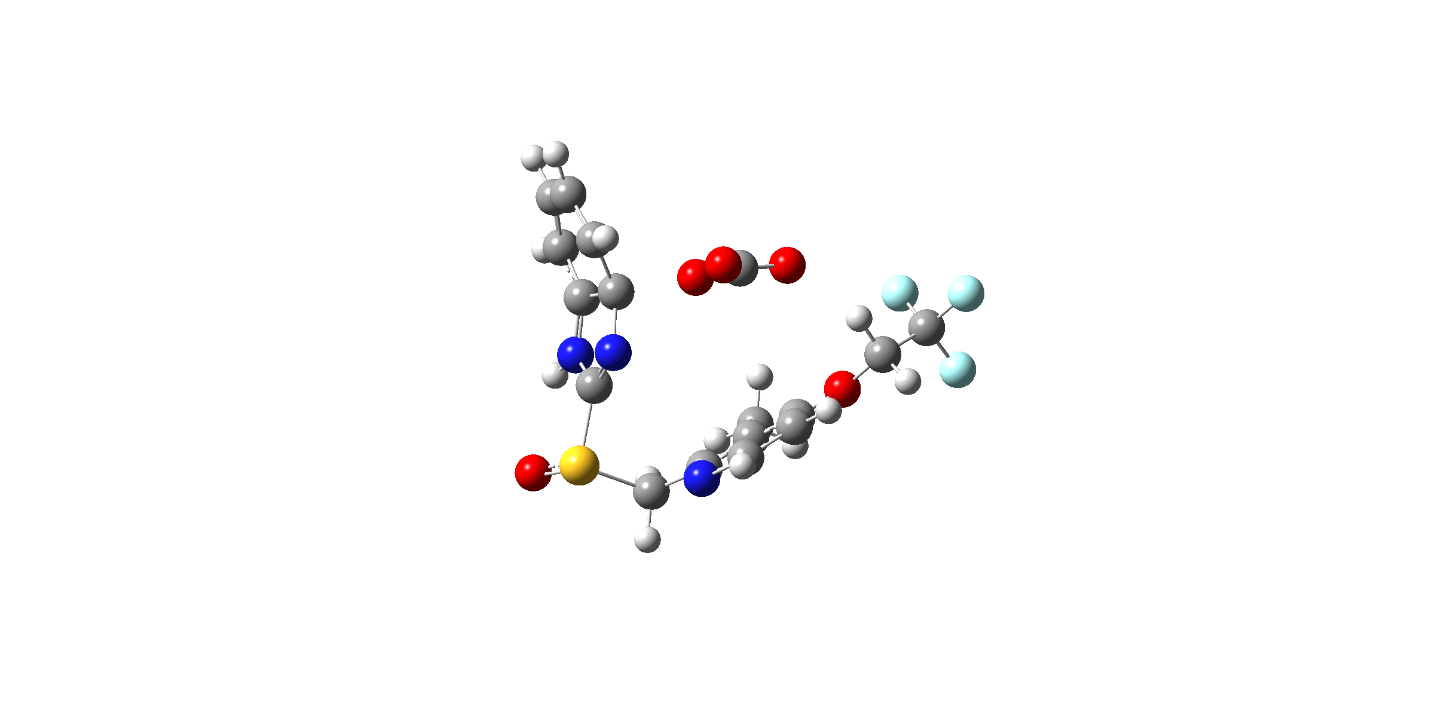


**Fig. S3.**

***** Axes restored to original set *****

orientation:

---------------------------------------------------------------------

Center Atomic Atomic Coordinates (Angstroms)

Number Number Type X Y Z

---------------------------------------------------------------------

1 6 0 -3.621171 3.494861 -0.861378

2 6 0 -3.316650 3.577174 0.555566

3 6 0 -2.704910 2.572956 1.210776

4 6 0 -2.193305 1.409499 0.398066

5 6 0 -2.948853 1.255597 -0.917887

6 6 0 -3.440264 2.367013 -1.620531

7 7 0 -2.228722 0.046644 0.992024

8 6 0 -2.636277 -0.707051 0.066037

9 7 0 -3.064586 -0.038498 -1.126868

10 16 0 -2.931008 -2.513388 0.119900

11 8 0 -3.959425 -2.707775 -0.982490

12 6 0 -1.300483 -3.064627 -0.617091

13 6 0 -0.120662 -2.512683 0.130499

14 6 0 0.912142 -1.813379 -0.499719

15 6 0 1.935776 -1.323096 0.339175

16 6 0 1.875987 -1.525402 1.714221

17 6 0 0.795588 -2.243981 2.217008

18 7 0 -0.179588 -2.737003 1.459151

19 6 0 1.010774 -1.533980 -1.976664

20 8 0 2.943520 -0.677093 -0.296678

21 6 0 3.831026 0.126799 0.467927

22 6 0 4.805972 0.742033 -0.512367

23 9 0 4.194792 1.497115 -1.444530

24 9 0 5.672965 1.535009 0.152622

25 9 0 5.528521 -0.190438 -1.167618

26 1 0 -4.089551 4.356544 -1.323907

27 1 0 -3.614705 4.474789 1.081893

28 1 0 -2.458949 2.613792 2.259448

29 1 0 -3.798857 2.290434 -2.637653

30 1 0 -3.513322 -0.515660 -1.904203

31 1 0 -1.365203 -4.149737 -0.515278

32 1 0 -1.363236 -2.798560 -1.668331

33 1 0 2.632449 -1.148101 2.387529

34 1 0 0.720463 -2.426550 3.284591

35 1 0 1.087429 -0.457480 -2.148396

36 1 0 1.915346 -1.985406 -2.393251

37 1 0 0.159341 -1.910881 -2.537848

38 1 0 3.278738 0.928138 0.964125

39 1 0 4.405791 -0.463736 1.185452

40 8 0 1.643422 2.083724 0.217915

41 6 0 0.578137 1.999620 0.813296

42 8 0 0.201167 2.073688 1.974300

43 8 0 -0.409418 1.765841 -0.103145

---------------------------------------------------------------------
